# Molecular Evolution of Trehalose-6-Phosphate Synthase (TPS) Gene Family in *Populus*, *Arabidopsis* and Rice

**DOI:** 10.1371/journal.pone.0042438

**Published:** 2012-08-08

**Authors:** Hai-Ling Yang, Yan-Jing Liu, Cai-Ling Wang, Qing-Yin Zeng

**Affiliations:** 1 College of Life Sciences and Biotechnology, Beijing Forestry University, Beijing, China; 2 State Key Laboratory of Systematic and Evolutionary Botany, Institute of Botany, Chinese Academy of Sciences, Beijing, China; University of Georgia, United States of America

## Abstract

Trehalose-6-phosphate synthase (TPS) plays important roles in trehalose metabolism and signaling. Plant TPS proteins contain both a TPS and a trehalose-6-phosphate phosphatase (TPP) domain, which are coded by a multi-gene family. The plant *TPS* gene family has been divided into class I and class II. A previous study showed that the *Populus*, *Arabidopsis*, and rice genomes have seven class I and 27 class II *TPS* genes. In this study, we found that all class I *TPS* genes had 16 introns within the protein-coding region, whereas class II *TPS* genes had two introns. A significant sequence difference between the two classes of TPS proteins was observed by pairwise sequence comparisons of the 34 TPS proteins. A phylogenetic analysis revealed that at least seven *TPS* genes were present in the monocot–dicot common ancestor. Segmental duplications contributed significantly to the expansion of this gene family. At least five and three *TPS* genes were created by segmental duplication events in the *Populus* and rice genomes, respectively. Both the TPS and TPP domains of 34 *TPS* genes have evolved under purifying selection, but the selective constraint on the TPP domain was more relaxed than that on the TPS domain. Among 34 *TPS* genes from *Populus*, *Arabidopsis*, and rice, four class I *TPS* genes (*AtTPS1*, *OsTPS1*, *PtTPS1*, and *PtTPS2*) were under stronger purifying selection, whereas three *Arabidopsis* class I *TPS* genes (*AtTPS2*, *3*, and *4*) apparently evolved under relaxed selective constraint. Additionally, a reverse transcription polymerase chain reaction analysis showed the expression divergence of the *TPS* gene family in *Populus*, *Arabidopsis*, and rice under normal growth conditions and in response to stressors. Our findings provide new insights into the mechanisms of gene family expansion and functional evolution.

## Introduction

Trehalose (α-D-glucopyranosyl α-D-glucopyranoside) is a non-reducing disaccharide in which two glucose units are linked in an α,α-1,1-glycosidic linkage. Trehalose plays important roles in protecting plants from heat, cold, and osmotic and dehydration stress [1], [2], [3], [4]. The biosynthesis of plant trehalose consists of two enzymatic steps. In the first, trehalose-6-phosphate synthase (TPS) catalyses the transfer of glucose from UDP-glucose to glucose 6-phosphate (G6P) to produce trehalose-6-phosphate (T6P). Subsequently, T6P is dephosphorylated into trehalose by trehalose-6-phosphate phosphatase (TPP). The plant TPS proteins contain the TPS and TPP domains, whereas TPP proteins contain only TPP domains. Plant TPP proteins have TPP activities [5]. However, although plant TPS proteins contain TPP domains, many studies have not detected TPP activity [6], [7]. The TPP domains in plant TPS proteins appear to have lost enzymatic activity during evolution. The evolutionary basis of this loss is not yet understood.

Each of the *Arabidopsis* and rice genomes has 11 *TPS* genes, and *Populus* contains 12 *TPS* genes [6], [7], [8]. These *TPS* genes have been divided into class I and class II [6], [7], [8]. Many studies have shown functional divergence among members of this gene family. Among the 11 rice *TPS* genes, functional complement assays performed in yeast *tps1* and *tps2* mutants revealed that only *OsTPS1* encodes an active TPS enzyme and that no OsTPS protein possesses TPP activity [7]. Among four *Arabidopsis* class I *TPS* genes, only AtTPS1 has TPS enzymatic activity, and no protein has significant TPP activity [6]. The *AtTPS1* gene plays important roles in the control of the stress response, cell and embryonic development, glucose sensing, and starch synthesis [9], [10], [11]. None of the seven *Arabidopsis* class II *TPS* genes (*AtTPS5–11*) complements the yeast *tps1* or *tps2* mutant phenotypes, suggesting that they are not actively or directly involved in trehalose metabolism [12]. But another study showed that *AtTPS6* has TPS and TPP enzymatic activities and can regulate cell shape and plant architecture [13]. Transgenic *Arabidopsis* plants harbouring the promoter-GUS/GFP protein reporters show that *Arabidopsis* class II *TPS* genes have remarkably different tissue-specific expression patterns.

The plant *TPS* gene family is a large gene family with multiple copies, and the members of this large gene family show extensive functional diversification [6], [8], [12], [13], [14]. In particular, class I and class II *TPS* genes show distinct characteristics in copy number, gene expression patterns, and enzyme and physiological functions. But it is still unclear what evolutionary mechanisms drive this functional divergence. In this study, we mainly focused on the following evolutionary questions. (1) Which genetic mechanisms contribute to the expansion of this gene family? (2) Do the TPS and TPP domains of *TPS* genes undergo similar selection pressure? (3) Which factors drive the functional divergence of class I and class II *TPS* genes? In order to address these questions, in this study, we examined the evolutionary characterisation of the *TPS* gene family in *Populus*, *Arabidopsis*, and rice. *Arabidopsis thaliana* is an important model for flowering plants (particularly eudicots), and rice (monocotyledon) is one of the most important food crops in the world. Perennial *Populus* is the most important model tree system of plant genomics currently available. These three angiosperm plants have completely sequenced and well-annotated genomes and have undergone at least one round of genome-wide duplication. Through a comprehensive analysis of gene sequences, gene structures, molecular evolution, and gene expression patterns, we provide a useful framework for further functional characterisation of the plant *TPS* gene family.

## Results

### Phylogenetic Analyses and Gene Structures of the *TPS* Gene Family in *Populus*, *Arabidopsis*, and Rice

**Figure 1:**
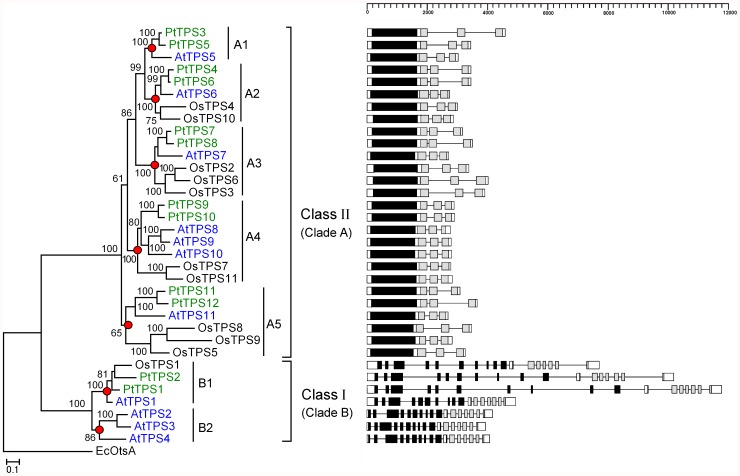
Phylogenetic relationships (A) and gene structures (B) of the*TPS* genes from *Populus*, *Arabidopsis,* and rice. Red circles indicate the most recent common ancestral *TPS* genes among *Populus*, *Arabidopsis,* and rice. Only bootstrap values >50% are shown. The TPS and TPP domains are highlighted by the black and grey boxes, respectively.

Previous studies identified 12 *TPS* genes in the *Populus* genome and 11 in each of the *Arabidopsis* and rice genomes (Table S1); these *TPS* genes are divided into class I and class II subfamilies [6], [7], [8]. *Populus*, *Arabidopsis*, and rice contain two (*PtTPS1* and *PtTPS2*), four (*AtTPS1*, *2*, *3*, and *4*), and one (*OsTPS1*) class I *TPS* genes, respectively, and the remaining 27 *TPS* genes are class II *TPS* genes 6,7,8. In this study, we constructed a phylogenetic tree of the 34 *TPS* genes from *Populus*, *Arabidopsis*, and rice (Figure 1A). The phylogenetic tree showed that the 34 *TPS* genes were divided into distinct two clades (clades A and B) with 100% bootstrap support. Clades A and B contained 27 class II and seven class I *TPS* genes, respectively. The seven class I *TPS* genes were further divided into two subclades (clades B1 and B2) with high bootstrap support. Clade B1 contained four class I *TPS* genes (*AtTPS1*, *OsTPS1*, *PtTPS1*, and *PtTPS2*) from *Populus, Arabidopsis*, and rice, whereas clade B2 contained only three *Arabidopsis* class I *TPS* genes (*AtTPS2*, *3*, and *4*).

We identified the nodes that lead to rice-specific and *Populus*–*Arabidopsis-*specific subclades (red circles in Figure 1A). These nodes represented the most recent common ancestral genes before the monocot and dicot split. The subclades defined by such nodes were designated as orthologous groups. Interestingly, subclade A1 only contained *Populus* and *Arabidopsis* class II *TPS* genes, indicating that genes may have been lost from the rice genome. Subclade B2 only contained three *Arabidopsis* class I *TPS* genes. We predicted that the *TPS* genes in subclade B2 might exist in the monocot–dicot common ancestor and were subsequently lost in *Populus* and rice. The number of subclades indicated that there were at least seven ancestral *TPS* genes in the monocot–dicot common ancestor. To further validate this conclusion, we performed a joint phylogenetic analysis with the *TPS* genes from *Populus*, *Arabidopsis*, rice, and other basal angiosperm species. To date, no complete genome sequence is available for basal angiosperm species. The most comprehensive genomic resources are the *Amborella* Genome Database (http://amborella.huck.psu.edu/) and Ancestral Angiosperm Genome Project (http://ancangio.uga.edu/). From these two databases, 15 *TPS* genes from *Amborella trichopoda*, *Persea americana*, *Liriodendron tulipifera*, *Aristolochia fimbriata*, and *Nuphar advena* were identified using TBLASTN searches. A phylogenetic tree with 49 *TPS* genes from *Populus*, *Arabidopsis*, rice, and five basal angiosperm species supported that there were at least seven ancestral *TPS* genes in the monocot–dicot common ancestor (red circles in Figure S1).

*TPS* genomic sequences were retrieved from the *Populus*, *Arabidopsis*, and rice genomes based on available information to investigate the intron/exon structures of the *TPS* genes. We found that all class I *TPS* genes had 16 introns within the protein-coding region by sequence comparisons between cDNAs and genomic DNA, whereas class II *TPS* genes had two introns (Figure 1B). The contrast in gene structures between class I and class II *TPS* members suggested their evolutionary divergence.

### Structural Features of *TPS* Proteins

**Figure 2:**
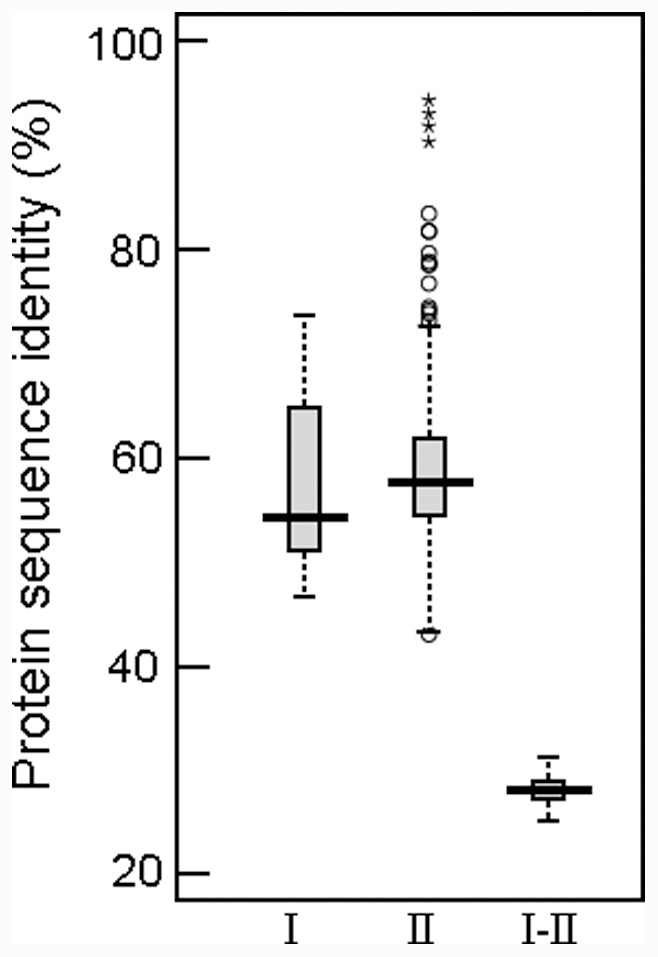
Pairwise sequence identity of full-length TPS proteins. I and II represent pairwise sequence identities of classes I and II TPS proteins, respectively. I–II represent pairwise sequence identities between class I and II TPS proteins. The boxplot shows the median (black line), interquartile range (box), and maximum and minimum scores (whiskers) for each data set. Outliers are shown as circles, and asterisks are outside of the whiskers.

Pairwise comparisons of the 34 TPS full-length protein sequences revealed some notable features (Figure 2). Seven class I TPS proteins showed 46.7–78.0% pairwise sequence identity. Twenty-seven class II TPS proteins had 43.1–94.4% pairwise sequence identity. But the protein sequences between the two TPS classes were significantly different (independent-sample *t*-test, *P*<0.0001) and only had 25.3–30.5% pairwise sequence identity.

**Figure 3:**
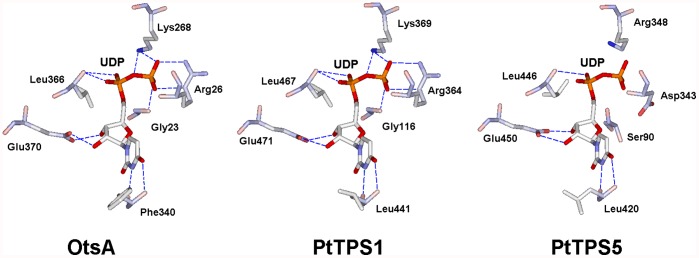
Schematic diagram of the interactions between UDP-glucose and TPS proteins.

The crystal structures of the *Escherichia coli* TPS protein (OtsA) have been resolved [15], which facilitated our understanding of the structural features of *TPS* family proteins. In this study, we modelled the three-dimensional (3D) structures of PtTPS1 and PtTPS5, representing two different classes of TPS proteins, based on the known 3D structure of the OtsA protein. TPS is a UDP-glucose (UDP-Glc)-dependent glycosyltransferase. It catalyses the synthesis of T6P using UDP-Glc as the donor and G6P as the acceptor. The X-ray structure of OtsA showed six key catalytic residues that could interact with the UDP-Glc substrate (Figure 3) [15]. Sequence comparisons showed that five sites among the six key catalytic residues were absolutely conserved in the seven class I TPS proteins, whereas only one site was absolutely conserved in the 27 class II TPS proteins (Figure S2). The predicted 3D structure of PtTPS1 showed that six residue sites could interact with the UDP-Glc substrate (Figure 3). However, only three sites in the PtTPS5 protein could interact with the UDP-Glc substrate (Figure 3). Thus, low pairwise sequence identity and a structural difference between the two classes of TPS proteins suggested their functional divergence.

### Chromosomal Locations and Gene Duplications in the *TPS* Gene Family of *Populus*, *Arabidopsis,* and Rice

**Figure 4:**
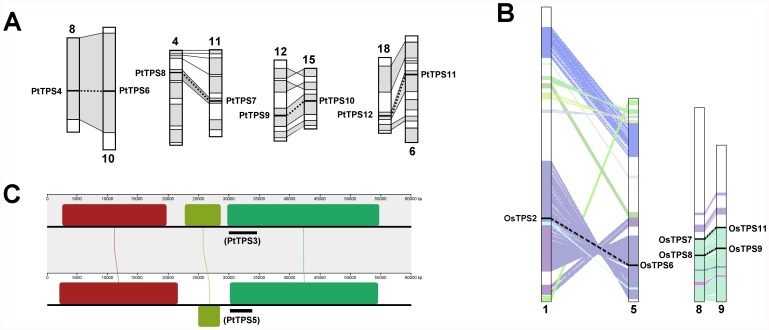
Duplicate gene pairs created by segmental duplication in *Populus* (A) and rice (B). Comparisons of the 60-kb flanking genomic regions of the paralogous gene pair *PtTPS3/5* (C). In (A), homologous genome blocks are shaded with grey and connected with lines. In (B), homologous genome blocks are shaded with colour. In (C), genome sequences were aligned using the progressive Mauve algorithm of Mauve v2.3.1 [29] with default parameters. Locally collinear blocks are indicated as same-coloured boxes and connected by thin coloured lines.

The physical locations of 10 among 12 *TPS* genes were assigned to nine *Populus* chromosomes, whereas the other two were assigned to two unattributed scaffold fragments. All 12 *TPS* genes were dispersed in *Populus* chromosomes (Figure S3). A phylogenetic analysis of the *TPS* gene family from *Populus*, *Arabidopsis*, and rice showed that *Populus* contained five recently duplicated gene pairs (*PtTPS3*/*5*, *PtTPS4*/*6*, *PtTPS7*/*8*, *PtTPS9*/*10*, and *PtTPS11*/*12*) (Figure 1A). A previous analysis of the *Populus* genome identified paralogous segments created by the whole-genome duplication event in the Salicaceae (salicoid duplication), which occurred 60–65 million years ago [16]. In this study, we found four duplicate pairs (*PtTPS4*/*6*, *PtTPS7*/*8*, *PtTPS9*/*10*, and *PtTPS11*/*12*) that were each located in a pair of paralogous blocks (Figure 4A), which indicated that the four duplicated gene pairs were created by a whole-genome duplication event. Additionally, comparisons of the 60-kb flanking genomic regions of the paralogous gene pair *PtTPS3/5* using Mauve software showed that this duplicate gene pair was formed by a segmental duplication event (Figure 4C).

In rice, all 11 *TPS* genes were dispersed in chromosomes 1, 2, 3, 5, 8, and 9 (Figure S3). The maximum-likelihood (ML) tree in Figure 1A shows that the rice genome contained four rice-specific duplicated gene pairs (*OsTPS2*/*6*, *OsTPS4*/*10*, *OsTPS7*/*11*, and *OsTPS8*/*9*). Segmental duplicated blocks have been identified in the rice genome (http://rice.plantbiology.msu.edu/). We found that three duplicated gene pairs (*OsTPS2*/*6*, *OsTPS7*/*11*, and *OsTPS8*/*9*) were each located in a pair of paralogous blocks (Figure 4B), indicating that these three duplicated gene pairs were formed by a segmental duplication event.

In the *Arabidopsis* genome, except for *AtTPS2* and *AtTPS3*, which were tandemly arrayed in chromosome 1, the other nine *TPS* genes were dispersed in chromosomes 1, 2, and 4 (Figure S3). The ML tree in Figure 1A shows that the *Arabidopsis* genome contained two *Arabidopsis*-specific duplicated gene pairs (*AtTPS2*/*3* and *AtTPS7/8*). *AtTPS7* and *AtTPS8* were created by a recent *Arabidopsis* whole-genome duplication event [17]. A previous co-linearity analysis suggested that the *AtTPS2* and *AtTPS3* genes arose from segmental duplication of the *AtTPS1* gene region, followed by a tandem duplication giving rise to the *AtTPS2* and *AtTPS3* gene pair [8].

### Molecular Evolution Analyses

**Figure 5:**
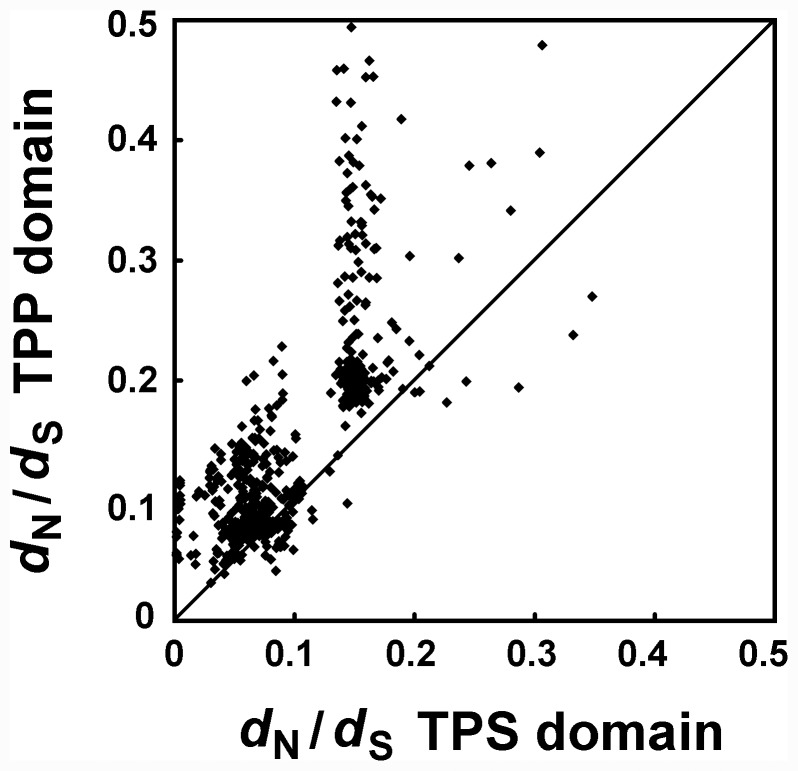
*d*_N_/*d*_S_ plot for the TPP domain versus the TPS domain of each pair of 34 *TPS* genes. These *TPS* genes were from *Populus*, *Arabidopsis,* and rice.

The ratio (ω) of the synonymous substitution rate (*d*_S_) versus the non-synonymous substitution rate (*d*_N_) provides a sensitive measure of selective pressure acting on a protein-coding gene. Homologous genes with ω ratios of 1, <1, or >1 are usually assumed to be evolving under neutral evolution, purifying selection, or positive selection, respectively. Plant TPS proteins consist of an N-terminal TPS domain and a C-terminal TPP domain [3]. To test for deviations in the substitution pattern of the two domains, we partitioned the 34 TPS sequences into TPS domain and TPP domain regions. The ω values were calculated across all pairwise comparisons within each of the 34 *TPS* genes using the YN00 program in the PAML software package [18]. A plot of *d*_N_/*d*_S_ for the TPS versus TPP domains is shown in Figure 5. The results suggested that both domains evolved under purifying selection but that the selective constraint on the TPP domain was more relaxed than that on the TPS domain (*t*-tests, *P*<0.0001).

**Figure 6:**
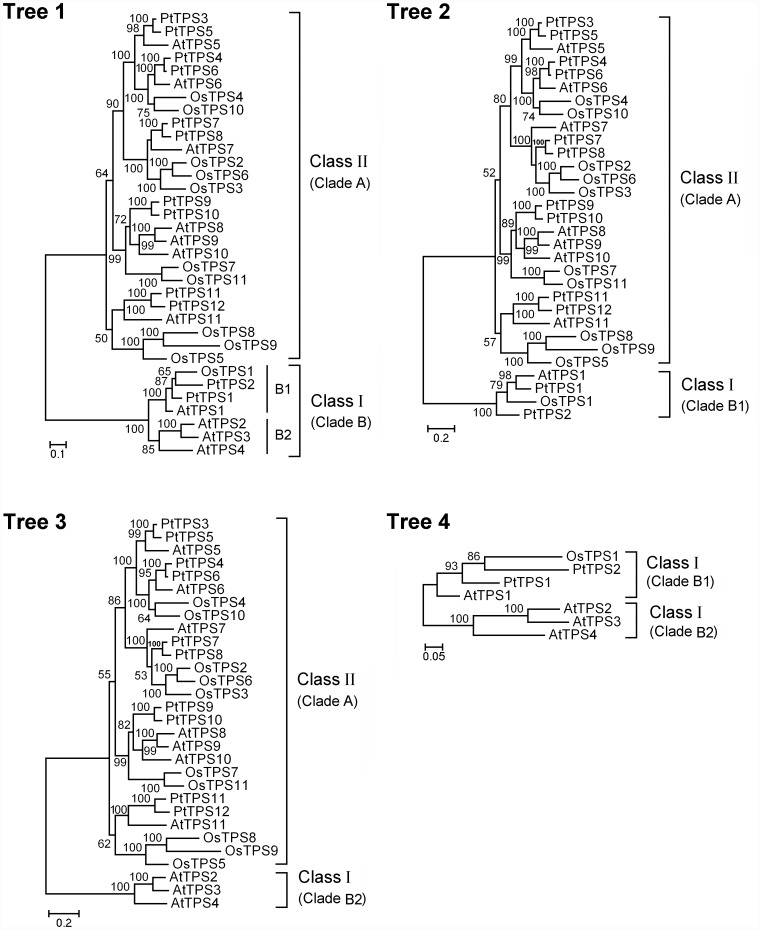
Phylogenetic trees used for molecular evolution analyses. The trees were reconstructed using a maximum-likelihood (ML) procedure with the JTT model and 100 bootstrap replicates. Only bootstrap values >50% are shown.

The *TPS* gene family in *Populus*, *Arabidopsis*, and rice was divided into two subfamilies (class I and class II) (Figure 1A). The *TPS* genes within the same class showed conserved gene structures and high protein sequence similarity, but gene structural variation and low protein sequence similarity were observed between the two classes of *TPS* genes. To test whether there were changes in selective pressure between the two classes of *TPS* genes, we conducted two branch-specific models (one-ratio model and two-ratio model) using the CODEML program in the PAML software package. The one-ratio model assumed a single ω ratio for *TPS* genes in the tree. This was compared using a likelihood ratio test (LRT) with the two-ratio model, which assumed different ω ratios in the two *TPS* genes clades. The test was conducted independently for the full-length gene, TPS, and TPP domain regions on the four unrooted trees (Figure 6). The comparison of the one-ratio model to the two-ratio model using LRTs showed that the two-ratio model was a significantly better fit than the one-ratio model for the full-length gene, TPS, and TPP domains (*P*<0.002) (tree 1 in Table 1), suggesting significant differences in selective pressures between the two classes of *TPS* genes. The mean ω value in the class II *TPS* genes was lower than that in the class I *TPS* genes (Table 1), indicating that the class II *TPS* genes (clade A in tree 1, Figure 6) were under stronger purifying selection than the class I *TPS* genes (clade B in tree 1, Figure 6). Class I *TPS* genes were further divided into two subclasses: clade B1 and B2 (tree 1 in Figure 6). We further tested whether there were changes in selective pressures between the class II *TPS* genes and class I *TPS* genes in clade B1 or clade B2. The PAML analysis showed that the class I *TPS* genes in clade B1 were under stronger purifying selection than the class II *TPS* genes in clade A for the full-length *TPS* genes and the TPS domain regions (*P*<0.001) (tree 2 in Table 1). However, the selective pressure for the TPP domain was not significantly different between the two clades. This indicated that the TPS domain contributed to the selective pressure change between the *TPS* genes in clades A and B1 (tree 2 in Figure 6). Clade B2 only contained three *Arabidopsis* class I *TPS* genes (*AtTPS2*, *3*, and *4*). The PAML analysis showed that three *Arabidopsis* class I *TPS* genes in clade B2 were under more relaxed selection pressure than the class II *TPS* genes in clade A in the full-length gene, the TPS domain, and the TPP domain regions (*P*<0.001) (tree 3 in Table 1). Additionally, the PAML analysis also showed that the three *Arabidopsis* class I *TPS* genes in clade B2 were under more relaxed selection pressure than the four class I *TPS* genes in clade B1 (*P*<0.001) (tree 4 in Table 1). Taken together, these results reveal that four class I *TPS* genes (*AtTPS1*, *OsTPS1*, *PtTPS1*, and *PtTPS2*) were under stronger purifying selection among 34 *TPS* genes from *Populus*, *Arabidopsis*, and rice, whereas three *Arabidopsis* class I *TPS* genes (*AtTPS2*, *3*, and *4*) apparently evolved under relaxed selective constraint.

**Table 1:** Summary statistics for detection of selection using branch specific models of PAML.

Tree	Model	Estimates of parameters	ln *L*	2 *Δl*	*P*
Tree 1					
Full length gene	One ratio	ω = 0.09382 for all branches	−47300.58064		
	Two ratios	ω_1_ = 0.08476 for clade A	−47274.88473	51.39182	<0.0001
		ω_0_ = 0.13127 for clade B			
TPS domain	One ratio	ω = 0.07925 for all branches	−28400.95963		
	Two ratios	ω_1_ = 0.07222 for clade A	−28388.21796	25.48336	<0.0001
		ω_0_ = 0.10836 for clade B			
TPP domain	One ratio	ω = 0.10915 for all branches	−14778.40868		
	Two ratios	ω_1_ = 0.10133 for clade A	−14773.41535	9.98666	0.0017
		ω_0_ = 0.14354 for clade B			
Tree 2					
Full length gene	One ratio	ω = 0.08217 for all branches	−45848.25709		
	Two ratios	ω_1_ = 0.08778 for clade A	−45829.27108	37.97203	<0.0001
		ω_0_ = 0.05162 for clade B1			
TPS domain	One ratio	ω = 0.06743 for all branches	−27558.71280		
	Two ratios	ω_1_ = 0.07500 for clade A	−27529.46938	58.48685	<0.0001
		ω_0_ = 0.03016 for clade B1			
TPP domain	One ratio	ω = 0.09627 for all branches	−13788.87778		
	Two ratios	ω_1_ = 0.09945 for clade A	−13787.24660	3.26236	
		ω_0_ = 0.07508 for clade B1			
Tree 3					
Full length gene	One ratio	ω = 0.09565 for all branches	−42556.07775		
	Two ratios	ω_1_ = 0.08393 for clade A	−42442.09292	227.96967	<0.0001
		ω_0_ = 0.35356 for clade B2			
TPS domain	One ratio	ω = 0.08229 for all branches	−25567.87248		
	Two ratios	ω_1_ = 0.07113 for clade A	−25487.99989	159.74518	<0.0001
		ω_0_ = 0.33343 for clade B2			
TPP domain	One ratio	ω = 0.10881 for all branches	−13198.32846		
	Two ratios	ω_1_ = 0.09997 for clade A	−13179.70794	37.24105	<0.0001
		ω_0_ = 0.30455 for clade B2			
Tree 4					
Full length gene	One ratio	ω = 0.12320 for all branches	−10909.79129		
	Two ratios	ω_1_ = 0.06510 for clade B1	−10798.89073	221.80112	<0.0001
		ω_0_ = 0.35030 for clade B2			
TPS domain	One ratio	ω = 0.09574 for all branches	−6148.55613		
	Two ratios	ω_1_ = 0.04285 for clade B1	−6063.71598	169.68030	<0.0001
		ω_0_ = 0.31121 for clade B2			
TPP domain	One ratio	ω = 0.13771 for all branches	−3405.43807		
	Two ratios	ω_1_ = 0.08930 for clade B1	−3387.03778	36.80058	<0.0001
		ω_0_ = 0.31125 for clade B2			

NOTE: All trees were shown in figure 6.

### Expression Patterns of *TPS* Gene Family in *Populus*, *Arabidopsis,* and Rice

**Figure 7:**
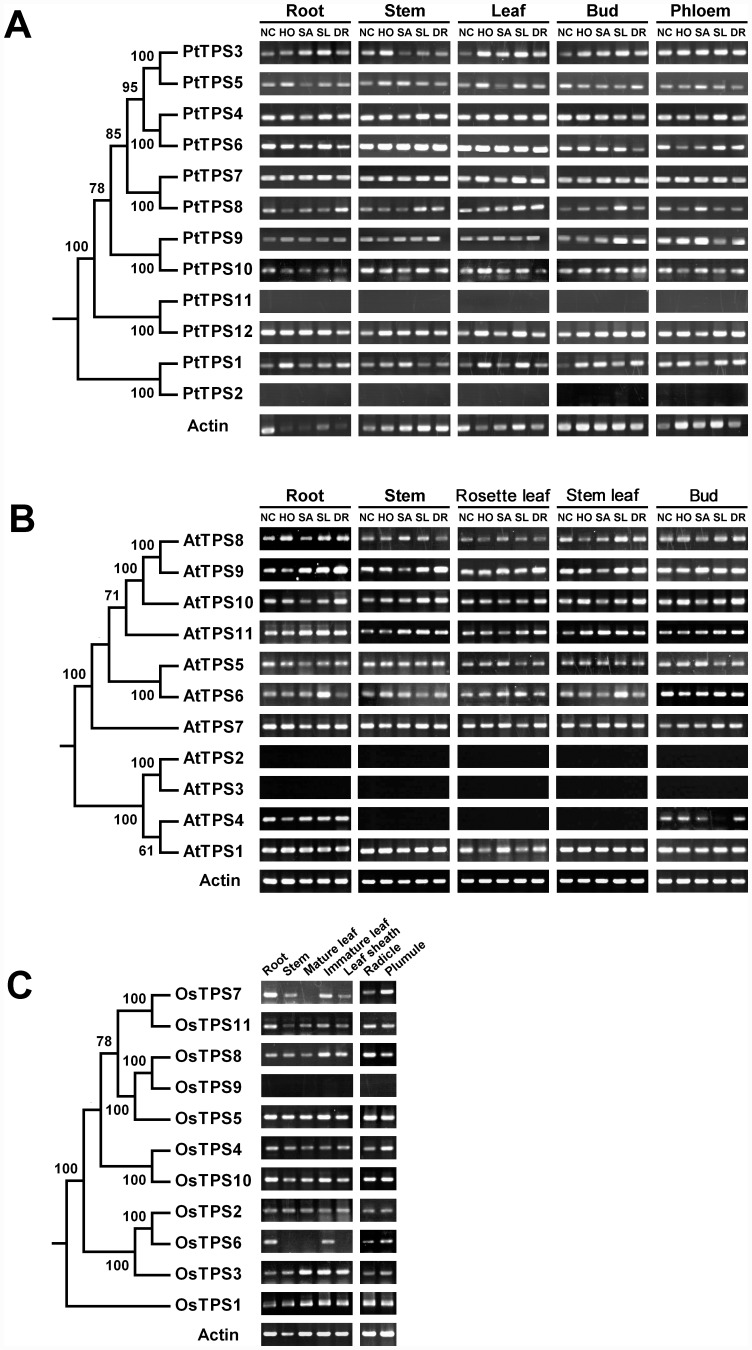
Expression patterns of *TPS* genes in *Populus* (A), *Arabidopsis* (B), and rice (C). NC indicates plants grown under normal growth conditions. HO, SA, SL, and DR indicate plants treated with H_2_O_2_, salicylic acid, NaCl, and drought stressors, respectively. Only bootstrap values >50% are shown in the phylogenetic trees.

The tissue-specific expression patterns of the *TPS* gene family in *Populus* and *Arabidopsis* were examined by reverse transcription polymerase chain reaction (RT-PCR) analysis under normal growth conditions and in response to stress treatments (H_2_O_2_, NaCl, salicylic acid, and drought). In *Populus*, ten *TPS* genes (*PtTPS1*, *3*, *4*, 5, *6*, *7*, *8*, *9*, *10*, and *12*) were expressed in all tissues under all growth conditions, whereas two *TPS* genes (*PtTPS2* and *PtTPS11*) were neither expressed in any tissue nor responded to any treatment applied in this study (Figure 7A). We did not find the *PtTPS2* EST sequence in the *Populus trichocarpa* expressed sequence tag (EST) database, but five *PtTPS11* ESTs (expressed in bud, floral bud, and leaf tissues) were identified (Table S2). Eight *Arabidopsis TPS* genes (*AtTPS1*, *5*, *6*, *7*, *8*, *9*, *10*, and *11*) were expressed in all the tissues examined, and two *TPS* genes (*AtTPS2* and *3*) were not expressed in any tissues examined (Figure 7B). *AtTPS4* was only expressed in root and flower bud tissues. In the *Arabidopsis thaliana* EST database, only a single *AtTPS2* EST (expressed in seeds) was identified, but we did not find the *AtTPS3* EST sequence (Table S2). In rice, we only analysed tissue-specific expression patterns of the *TPS* gene family under normal growth conditions. Eight rice *TPS* genes (*OsTPS1*, *2*, *3*, *4*, *5*, *8*, *10*, and *11*) were expressed in all tissues, whereas *OsTPS9* was not expressed in the tissues examined. *OsTPS6* and *OsTPS7* were selectively expressed in some specific tissues (Figure 7C). An EST search showed that *OsTPS9* was expressed in the panicle and pistil (Table S2). In this study, both the RT-PCR experiment and EST search did not identify expression of the *PtTPS2* and *AtTPS3* genes. These two genes might be expressed at sub-detectable levels, or they are only induced in response to treatments and/or in tissues not examined in our study, or they are pseudogenes.

## Discussion

The plant *TPS* genes play essential roles in the regulation of sugar metabolism, embryonic development, and response to abiotic stress. Plant *TPS* genes fall into two distinct classes. Many studies have shown distinct functional divergence between the two classes of *TPS* genes. Our molecular evolution analysis revealed that the TPS domains of four class I *TPS* genes (*AtTPS1*, *OsTPS1*, *PtTPS1*, and *PtTPS2*) among 34 *TPS* genes from *Populus*, *Arabidopsis*, and rice, were under stronger purifying selection, indicating functional conservation of the TPS domain among the four *TPS* genes. Sequence comparisons and predicted 3D structures also indicated that class I TPS proteins had TPS activity. Functional complement assays performed in yeast *tps1* and *tps2* mutants revealed that the OsTPS1 and AtTPS1 had TPS activities but no TPP activity [6], [7]. Thus, the results of the molecular evolution analysis were well supported by these experimental results. Sequence and structural analyses showed that class II TPS proteins might not interact with the UDP-Glc substrate, suggesting that class II TPS proteins are not directly involved in trehalose metabolism. However, many class II TPS genes were expressed in different tissues in *Populus*, *Arabidopsis*, and *rice*, suggesting that class II TPS genes might have new functions. For example, *Arabidopsis AtTPS5* plays a role in thermotolerance, possibly through its interaction with the transcriptional co-activator MBF1c [19]. Interestingly, three *Arabidopsis* class I *TPS* genes (*AtTPS2*, *3*, and *4*) apparently evolved under relaxed selective constraint, indicating functional divergence. Functional complement assays performed in yeast *tps1* and *tps2* mutants revealed that all AtTPS2, AtTPS3, and AtTPS4 had no TPS or TPP activity [6]. The possible molecular causes for the loss of AtTPS2 and AtTPS4 activity were considered to accumulate deleterious mutations and alter protein conformations [6]. Additionally, the expression patterns of *AtTPS2*, *AtTPS3* and *AtTPS4* had significant differences compared with *AtTPS1*. *AtTPS1* was expressed in roots, stems, rosette leaves, flower buds, and ripening siliques [20]. However, the expressions of *AtTPS2* and *AtTPS3* were not detected by RT-PCR in *Arabidopsis*. *AtTPS4* was only expressed in root and flower bud tissues. Other studies found that *AtTPS2* is expressed in seeds [21]. AtTPS2-specific GUS expression was detected only in the chalazal endosperm [6]. Thus, the molecular evolution analysis, gene expression patterns, and functional complement assays indicated functional divergence between the three *Arabidopsis* class I *TPS* genes and *AtTPS1*.

Gene duplication and subsequent functional divergence of the duplicate genes has been recognised as an important source of evolutionary novelty. Several possible evolutionary fates of duplicate genes have been proposed [22], [23], [24]: (1) non-functionalisation, in which one duplicate gene accumulates deleterious mutations as a pseudogene, whereas another duplicate gene maintains its original function; (2) neo-functionalisation, in which one duplicate copy accumulates beneficial mutations and acquires a new function, whereas another duplicate copy retains the original function; (3) sub-functionalisation, in which each descendant copy adopts some of the tasks of the ancestral gene. In this study, nine *TPS* duplicate pairs (*AtTPS8*/*9*, *PtTPS3*/*5*, *4*/*6*, *7*/*8*, *9*/*10*, and *11*/*12*, *OsTPS2*/*6*, *7/11*, and *8*/*9*) were created by segmental duplication events, and one duplicate pair, *AtTPS2*/*3*, was formed by tandem duplication. The gene expression-pattern analyses showed that two duplicate copies shared similar expression patterns in five duplicate gene pairs (*PtTPS3*/*5*, *PtTPS4*/*6*, *PtTPS7*/*8*, *PtTPS9*/*10, and AtTPS8*/*9*), suggesting that two duplicate genes had redundant functions or that functional divergence not identified in our study had occurred, such as protein function. For the duplicate pair *AtTPS2*/*3*, *AtTPS2* was expressed in seeds, whereas *AtTPS3* was not expressed in all tissues examined, suggesting that *AtTPS3* may have become a pseudogene or evolved a new function not identified in our study. One copy of each of four duplicate gene pairs (*PtTPS11*/*12*, *OsTPS2*/*6*, *OsTPS7/11*, and *OsTPS8*/*9*) was expressed in all tissues examined, whereas the other was expressed only in a specific tissue. This sub-functionalisation may be the evolutionary fate of the four duplicate gene pairs.

## Methods

### Phylogenetic Analyses

A previous study identified 12 *TPS* genes in the *Populus* genome (Build version 1.2) [8]. Eleven *TPS* genes were identified in each of the *Arabidopsis* and rice genomes [7], [25]. These TPS protein sequences were aligned using MUSCLE software [26] and further adjusted manually using BioEdit [27]. The phylogenetic relationships of the *Populus*, *Arabidopsis*, and rice *TPS* gene family were reconstructed based on full-length protein sequences using a ML procedure implemented in PHYML [28] with the JTT (Jones, Taylor & Thornton) amino acid substitution model. The *Escherichia coli* TPS protein (OtsA) was used as an out group in the phylogenetic analysis. Sequence comparisons between cDNA and genomic DNA were performed using BioEdit software and further adjusted manually using BioEdit [27].

### Homology Modelling

The crystal structures of the *Escherichia coli* TPS protein (Protein Data Bank code no.: 1GZ5) was used as a template for constructing structural models of PtTPS1 and PtTPS5. The sequences were aligned using the Align 2D structure alignment program (Homology Module in InsightII software; Accelrys, San Diego, CA, USA). Structures were automatically built using the modeller module of InsightII. All structures were verified by the profile-3D program in InsightII. The models were selected according to the model evaluation score calculated by profile-3D.

### Molecular Evolution Analyses

To evaluate variation in selective pressure in a phylogeny, the branch-specific models of CODEML in the software package PAML [18] were used to estimate ω under different assumptions. Analyses were conducted under two *a priori* assumptions: a one-ratio model in which one ω value was assumed for the entire tree, and a two-ratio model in which ω values were allowed to vary between the two different clades. To verify which of the models best fit the data, LRTs were performed by comparing twice the difference in log-likelihood values between pairs of the models using a χ2 distribution, with the degrees of freedom equal to the differences in the number of parameters between the models [18].

### Expression of *TPS* Genes in *Populus*, *Arabidopsis,* and Rice

Seedlings of *P. trichocarpa* (Torr. & Gray) were cultivated in potting soil for 2 months. *Populus* seedlings were separately irrigated and sprayed with 150 mM NaCl, 0.5% H_2_O_2_, or 3.5 mM salicylic acid solution. Each treatment was conducted with five replicate plants for 12 hr. Drought stress was evaluated by withholding water for 2 weeks. After the stress treatments, total RNA was isolated from leaf, shoot, bud, phloem, and root tissues of each seedling. The 4-week-old *Arabidopsis* plants (*Arabidopsis thaliana* ecotype Columbia-0) were transferred into a 250 mM NaCl solution for a 5 hr salt treatment, 1 mM H_2_O_2_ solution for 5 hr for the oxidative stress treatment, and Whatman filter paper for 2 hr for the drought-stress treatment, respectively. *Arabidopsis* plants were irrigated with 5 mM salicylic acid solution for 24 hours for the salicylic acid treatment. After the stress treatments, total RNA was isolated from root, stem, rosette leaf, cauline leaf, and flower bud tissues of each *Arabidopsis* plant. The seedlings of *Oryza sativa* L. ssp. japonica were grown individually in 10×10 cm pots for 3 months. Total RNA was isolated from mature leaf, immature leaf, leaf sheath, stem, and root tissues of each rice seedling. Total RNA was isolated using an Aurum Total RNA Kit (Bio-Rad Laboratories, Hercules, CA, USA). Then, total RNA was treated with RNase-free DNase I (Promega, Madison, WI, USA) and reverse transcribed into cDNA using an RNA PCR Kit (AMV) version 3.0 (TaKaRa Bio, Shiga, Japan). Thirty-four specific primer pairs were designed based on the multiple sequence alignment of the *TPS* sequences (Table S3). In all RT-PCR analyses, the *actin* gene was used as an internal control. PCR conditions were optimised to consist of an initial denaturation of 3 min at 95°C, followed by 35 cycles of 30 s at 94°C, 30 s at 60°C, and 30 s at 72°C, with a final extension of 5 min at 72°C. After RT-PCR, the PCR products from each sample were analysed on 1% agarose gels and validated by DNA sequencing. All RT-PCR results were obtained from three independent experiments. Expression patterns of the *TPS* genes in this study were also inferred from a BLAST analysis of the EST database at the National Centre for Biotechnology Information (NCBI) EST database. To date, the NCBI EST database has 1,529,700 *Arabidopsis thaliana* ESTs, 987,318 *Oryza sativa* japonica ESTs, and 89,943 *Populus trichocarpa* ESTs. A minimum cut-off E value (Δ<e−20) was applied to select significant matches. A threshold of at least 97% sequence identity was employed.

## Supporting Information

Figure S1 **Phylogenetic tree of** ***TPS*** **genes from** ***Populus***, ***Arabidopsis*** **, rice, and five basal angiosperm species.** Red circles indicate the most recent common ancestral *TPS* genes among *Populus*, *Arabidopsis,* and rice.
(TIF)

Figure S2 **Sequence alignment of TPS proteins from** ***Populus*** **,** ***Arabidopsis,*** **and rice.** Conserved residues in TPS proteins are shaded in black and gray. The key catalytic residues interacting with UDP-glucose in the crystal structure of *E. coli* OtsA are indicated by red arrows. Conserved key catalytic residues interacting with UDP-glucose are shaded in green.
(TIF)

Figure S3 **Genomic localisation of the** ***TPS*** **genes in** ***Populus*** **(A),** ***Arabidopsis*** **(B), and rice (C).**
(TIF)

Table S1 **The** ***TPS*** **genes used to reconstruct phylogenetic trees.**
(DOC)

Table S2 **The numbers of** ***TPS*** **ESTs identified from rice,** ***Arabidopsis*** **and** ***Populus*** **EST databases in NCBI.**
(DOC)

Table S3 **Primers used to detect the expression of** ***TPS*** **genes.**
(DOC)
