# Supplementary figures and images for: Molecular Evolution of Trehalose-6-Phosphate Synthase (TPS) Gene Family in Populus, Arabidopsis and Rice
Source: PLoS One. 2012 Aug 8;7(8):e42438. doi: 10.1371/journal.pone.0042438 (PMC3414516; doi:10.1371/journal.pone.0042438)

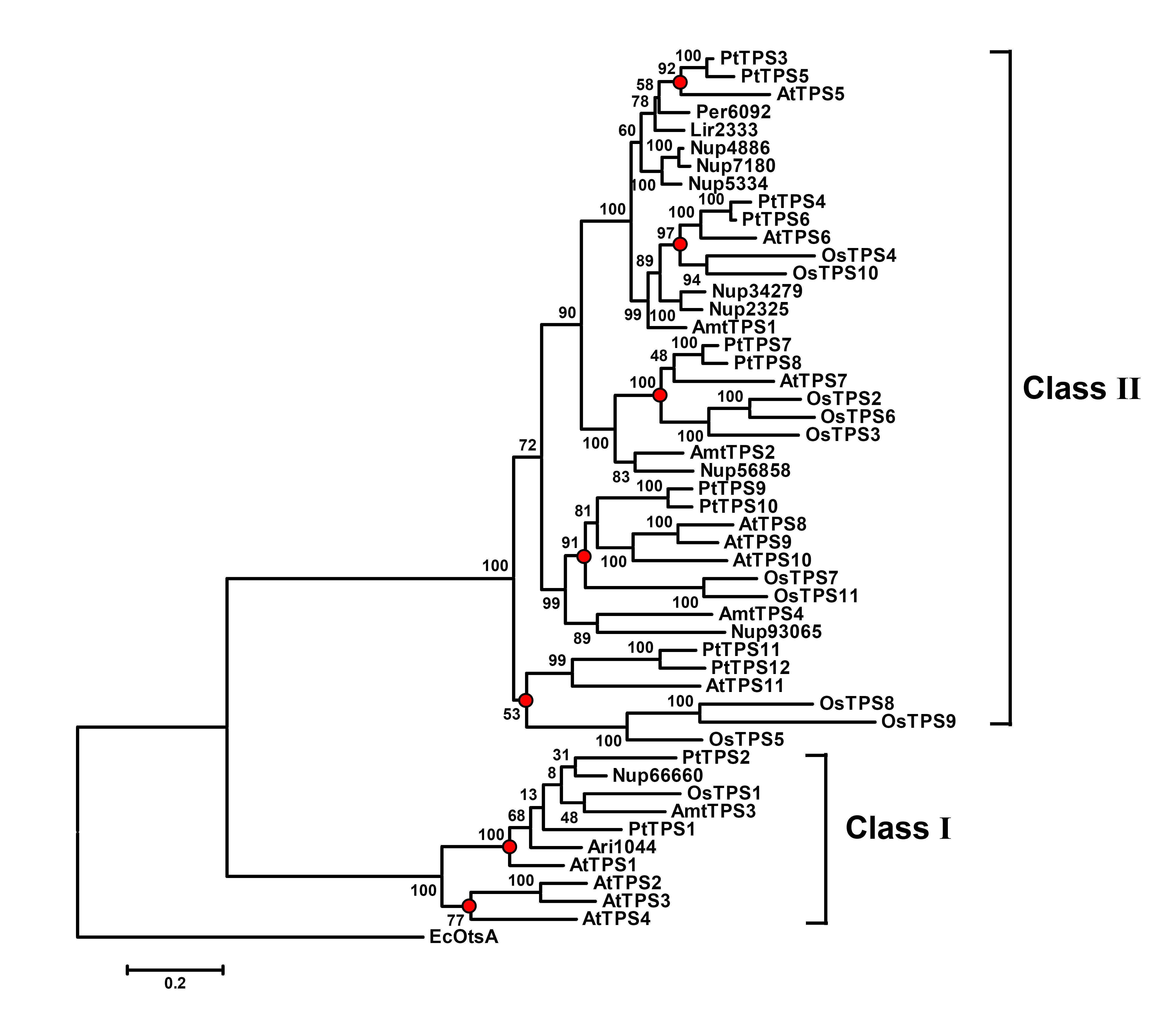

Supplement: Figure S1 — Phylogenetic tree of TPS genes from Populus, Arabidopsis , rice, and five basal angiosperm species. Red circles indicate the most recent common ancestral TPS genes among Populus, Arabidopsis, and rice. (TIF) [file pone.0042438.s001.tif]

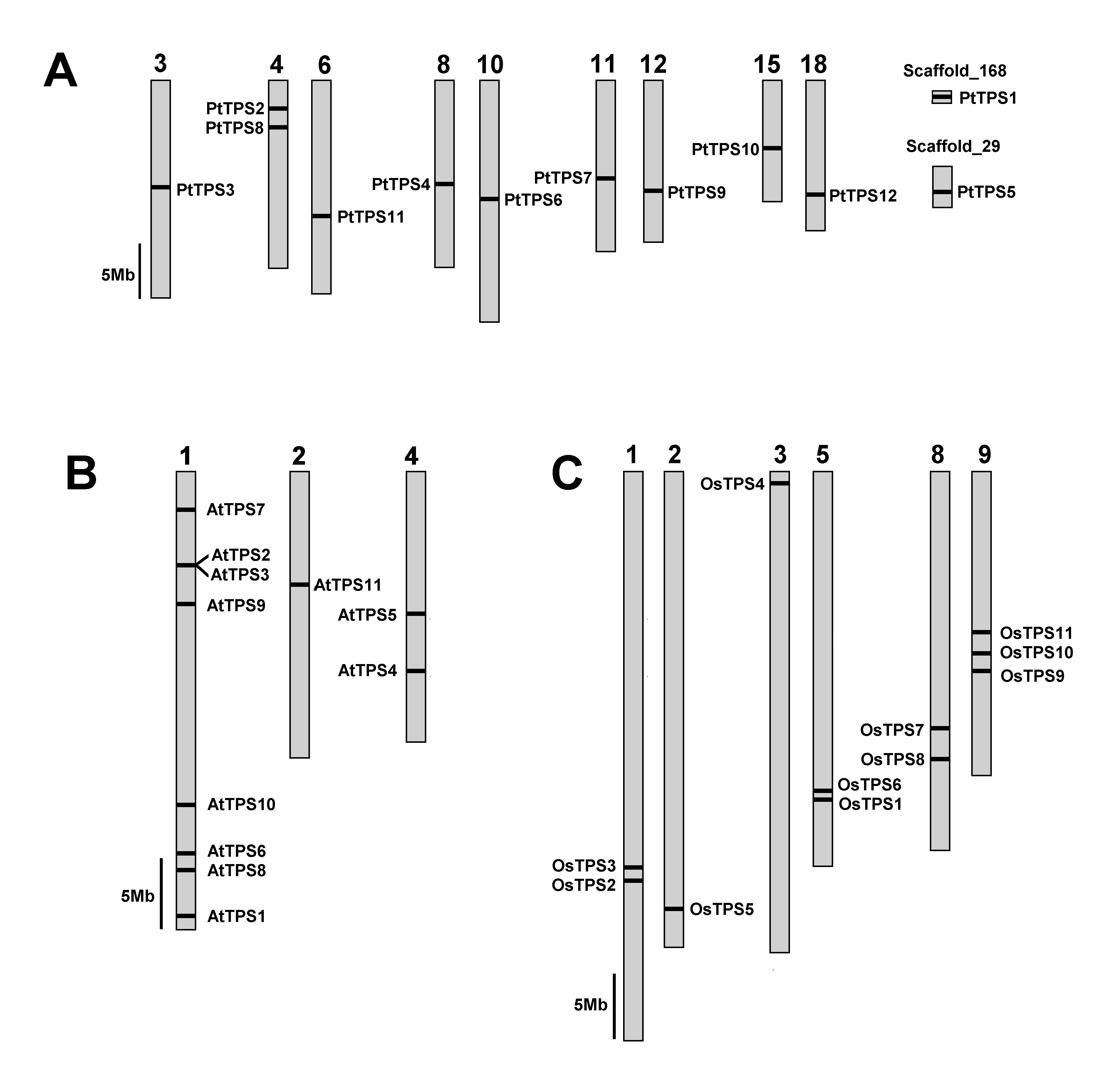

Supplement: Figure S3 — Genomic localisation of the TPS genes in Populus (A), Arabidopsis (B), and rice (C). (TIF) [file pone.0042438.s003.tif]
