# Supplementary material for: Molecular Evolution of Trehalose-6-Phosphate Synthase (TPS) Gene Family in Populus, Arabidopsis and Rice
Source: PLoS One. 2012 Aug 8;7(8):e42438. doi: 10.1371/journal.pone.0042438 (PMC3414516; doi:10.1371/journal.pone.0042438)
Supplement: Table S1 — The TPS genes used to reconstruct phylogenetic trees. (DOC) [file pone.0042438.s004.doc]

**Table S1.** The *TPS* genes used to reconstruct phylogenetic trees.

| Organism | Sequence name | Gene identifier | Database |
| --- | --- | --- | --- |
| *Arabidopsis thaliana* | AtTPS1 | At1g78580 | TAIR |
|  | AtTPS2 | At1g16980 |  |
|  | AtTPS3 | At1g17000 |  |
|  | AtTPS4 | At4g27550 |  |
|  | AtTPS5 | At4g17770 |  |
|  | AtTPS6 | At1g68020 |  |
|  | AtTPS7 | At1g06410 |  |
|  | AtTPS8 | At1g70290 |  |
|  | AtTPS9 | At1g23870 |  |
|  | AtTPS10 | At1g60140 |  |
|  | AtTPS11 | At2g18700 |  |
| *Oryza sativa* | OsTPS1 | LOC_Os05g44210 | MSU Rice Genome Annotation Project |
|  | OsTPS2 | LOC_Os01g54560 |  |
|  | OsTPS3 | LOC_Os01g53000 |  |
|  | OsTPS4 | LOC_Os03g12360 |  |
|  | OsTPS5 | LOC_Os02g54820 |  |
|  | OsTPS6 | LOC_Os05g44100 |  |
|  | OsTPS7 | LOC_Os08g31980 |  |
|  | OsTPS8 | LOC_Os08g34580 |  |
|  | OsTPS9 | LOC_Os09g25890 |  |
|  | OsTPS10 | LOC_Os09g23350 |  |
|  | OsTPS11 | LOC_Os09g20990 |  |
| *Populus trichocarpa* | PtTPS1 | estExt_fgenesh4_pg.C_1680018 | JGI Populus trichocarpa v1.1 |
|  | PtTPS2 | e_gw1.IV.2524.1 |  |
|  | PtTPS3 | fgenesh4_pg.C_LG_III000738 |  |
|  | PtTPS4 | grail3.0010065002 |  |
|  | PtTPS5 | estExt_Genewise1_v1.C_290287 |  |
|  | PtTPS6 | estExt_Genewise1_v1.C_LG_X6311 |  |
|  | PtTPS7 | eugene3.00110684 |  |
|  | PtTPS8 | fgenesh4_pg.C_LG_IV000367 |  |
|  | PtTPS9 | fgenesh4_pm.C_LG_XII000278 |  |
|  | PtTPS10 | eugene3.00150531 |  |
|  | PtTPS11 | eugene3.00061363 |  |
|  | PtTPS12 | fgenesh4_pm.C_LG_XVIII000320 |  |
| *Amborella trichopoda* | AmtTPS1 | GSVIVT01011634001|PACid:17823444 | Amborella Genome Database |
|  | AmtTPS2 | GSVIVT01020754001|PACid:17830047 |  |
|  | AmtTPS3 | GSVIVT01014092001|PACid:17825150 |  |
|  | AmtTPS4 | GSVIVT01007789001|PACid:17820521 |  |
| *Nuphar advena* | Nup5334 | Gnl|Nuphar|b3_c5334 | Ancestral Angiosperm Genome Project |
|  | Nup7180 | Gnl|Nuphar|b3_c7180 |  |
|  | Nup4886 | Gnl|Nuphar|b3_c4886 |  |
|  | Nup2325 | Gnl|Nuphar|b3_c2325 |  |
|  | Nup34279 | Gnl|Nuphar|b3_c34279 |  |
|  | Nup56858 | Gnl|Nuphar|b3_c56858 |  |
|  | Nup93065 | Gnl|Nuphar|b3_c93065 |  |
|  | Nup66660 | Gnl|Nuphar|b3_c66660 |  |
| *Aristolochia fimbriata* | Lir2333 | gnl|Liriodendron|b4_c2333 | Ancestral Angiosperm Genome |
| *Liriodendron tulipifera* | Ari1044 | gnl|Aristolochia|b3_c1044 | Ancestral Angiosperm Genome |
| *Persea americana* | Per6092 | gnl|Persea|b4_c6092 | Ancestral Angiosperm Genome |
