# Supplementary material for: Molecular Evolution of Trehalose-6-Phosphate Synthase (TPS) Gene Family in Populus, Arabidopsis and Rice
Source: PLoS One. 2012 Aug 8;7(8):e42438. doi: 10.1371/journal.pone.0042438 (PMC3414516; doi:10.1371/journal.pone.0042438)
Supplement: Table S2 — The numbers of TPS ESTs identified from rice, Arabidopsis and Populus EST databases in NCBI. (DOC) [file pone.0042438.s005.doc]

**Table S2**. The numbers of *TPS* ESTs identified from rice, *Arabidopsis* and *Populus* EST databases in NCBI.

|  |  | Tissues |  |  |  |  |  |  |  |
| --- | --- | --- | --- | --- | --- | --- | --- | --- | --- |
| Organisms | *TPS* genes | Root | Leaf | Shoot | Callus | Flower | Panicle | Anther | Pistil |
| *Oryza sativa* | *OsTPS1* | 0 | 2 | 2 | 3 | 1 | 0 | 0 | 0 |
|  | *OsTPS2* | 10 | 1 | 10 | 9 | 9 | 6 | 0 | 0 |
|  | *OsTPS3* | 0 | 0 | 2 | 7 | 1 | 3 | 6 | 0 |
|  | *OsTPS4* | 45 | 2 | 0 | 1 | 1 | 48 | 0 | 0 |
|  | *OsTPS5* | 62 | 19 | 1 | 3 | 0 | 3 | 2 | 0 |
|  | *OsTPS6* | 0 | 7 | 5 | 5 | 1 | 0 | 0 | 1 |
|  | *OsTPS7* | 1 | 2 | 0 | 8 | 0 | 0 | 0 | 0 |
|  | *OsTPS8* | 32 | 3 | 6 | 8 | 1 | 27 | 0 | 2 |
|  | *OsTPS9* | 0 | 0 | 0 | 0 | 0 | 96 | 0 | 4 |
|  | *OsTPS10* | 1 | 1 | 1 | 7 | 0 | 0 | 0 | 0 |
|  | *OsTPS11* | 1 | 0 | 1 | 2 | 0 | 0 | 0 | 0 |
|  |  |  |  |  |  |  |  |  |  |
|  |  | Root | Leaf | Ovule | Green silique | Rosette | Whole plant | Seed |  |
| *Arabidopsis thaliana* | *AtTPS1* | 1 | 0 | 5 | 0 | 0 | 6 | 3 |  |
|  | *AtTPS2* | 0 | 0 | 0 | 0 | 0 | 0 | 1 |  |
|  | *AtTPS4* | 0 | 0 | 1 | 0 | 0 | 1 | 0 |  |
|  | *AtTPS5* | 0 | 1 | 2 | 0 | 0 | 11 | 1 |  |
|  | *AtTPS6* | 0 | 0 | 2 | 0 | 1 | 9 | 0 |  |
|  | *AtTPS7* | 0 | 0 | 1 | 0 | 0 | 5 | 1 |  |
|  | *AtTPS8* | 1 | 5 | 0 | 1 | 0 | 12 | 0 |  |
|  | *AtTPS9* | 0 | 1 | 0 | 0 | 0 | 7 | 0 |  |
|  | *AtTPS10* | 5 | 1 | 2 | 0 | 0 | 8 | 1 |  |
|  | *AtTPS11* | 1 | 2 | 0 | 2 | 0 | 6 | 0 |  |
|  |  |  |  |  |  |  |  |  |  |
|  |  | Leaf | Bud | Floral bud | Outer xylem |  |  |  |  |
| *Populus trichocarpa* | *PtTPS1* | 0 | 0 | 5 | 4 |  |  |  |  |
|  | *PtTPS4* | 0 | 1 | 0 | 0 |  |  |  |  |
|  | *PtTPS5* | 0 | 0 | 0 | 1 |  |  |  |  |
|  | *PtTPS7* | 0 | 2 | 0 | 0 |  |  |  |  |
|  | *PtTPS11* | 2 | 2 | 1 | 0 |  |  |  |  |
