# Supplementary material for: Molecular Evolution of Trehalose-6-Phosphate Synthase (TPS) Gene Family in Populus, Arabidopsis and Rice
Source: PLoS One. 2012 Aug 8;7(8):e42438. doi: 10.1371/journal.pone.0042438 (PMC3414516; doi:10.1371/journal.pone.0042438)
Supplement: Table S3 — Primers used to detect the expression of TPS genes. (DOC) [file pone.0042438.s006.doc]

**Table S3.** Primers used to detect the expression of *TPS* genes.

| **Plants** | **Gene** | **Primer name** | **Sequence（5’-3’）** |
| --- | --- | --- | --- |
| *Populus* | *PtTPS1* | PtTPS1-SP1 | TGTACCTCCCAAAATGCCTTAAG |
|  |  | PtTPS1-SP2 | GTCAACACCTAGCATTACCTTTCG |
|  | *PtTPS2* | PtTPS2-SP1 | AGGCAAGATCAGAGGCTTCCT |
|  |  | PtTPS2-SP2 | GTTGAAAGGCGATCTTCTTGG |
|  | *PtTPS3* | PtTPS3-SP1 | TTTAGGTTTTCGAGTCATTGCCT |
|  |  | PtTPS3-SP2 | CCCATTCCACATCATGATTGG |
|  | *PtTPS4* | PtTPS4-SP1 | TGTTGAGGACAAAGAAACTGCAC |
|  |  | PtTPS4-SP2 | CGCATTTTGAGTAGCTGTCATAGA |
|  | *PtTPS5* | PtTPS5-SP1 | TATAAGAGGACCAAGAACCGAGTG |
|  |  | PtTPS5-SP2 | GAACTCTCTTTCGTCTCAATGGC |
|  | *PtTPS6* | PtTPS6-SP1 | CTCTGAGTGTGAGAAGCTGGGAT |
|  |  | PtTPS6-SP2 | AGCAATTGATGGGCCTGC |
|  | *PtTPS7* | PtTPS7-SP1 | CACCATCAATAAGACCCCAAACC |
|  |  | PtTPS7-SP2 | GCTCTCAAGATGGTCCAACAAT |
|  | *PtTPS8* | PtTPS8-SP1 | AGGTGGTCTGCAGATGATGTT |
|  |  | PtTPS8-SP2 | CAGATTTACTGGAGTTAAGGATACCAC |
|  | *PtTPS9* | PtTPS9-SP1 | AAACGAGATCCTTCGCTGCT |
|  |  | PtTPS9-SP2 | GTTTCCCATTAGCAATCATCGA |
|  | *PtTPS10* | PtTPS10-SP3 | AATGGTGCCTCATACTTCTCTTGCTAA |
|  |  | PtTPS10-SP4 | CCAATCAAAATCCACAGCCAAAGAA |
|  | *PtTPS11* | PtTPS11-SP1 | TCCTCTAAGCAAGTGGTTCTCC |
|  |  | PtTPS11-SP2 | TTGCTTACTCCCTGCTGTGGT |
|  | *PtTPS12* | PtTPS12-SP1 | GGTGTACACAGCGACAACTGATG |
|  |  | PtTPS12-SP2 | CGTAGCAAGGCCTTGAAGCA |
|  | *Actin* | PtActin-5 | GTGAGCAACTGGGATGACATG |
|  |  | PtActin-6 | TCATGATGGAGTTGTATGTGGTCT |
| *Arabidopsis* | *AtTPS1* | AtTPS1-SP1 | GATCGGTCTCTGGACTTTCATGCTT |
|  |  | AtTPS1-SP2 | CCAAAACACTTCTGCTGCTTCCG |
|  | *AtTPS2* | AtTPS2-SP1 | TCAACTACTTATGTGCAATATACGCAATTG |
|  |  | AtTPS2-SP2 | GCATCTCGGATTCAGGAATAATGC |
|  | *AtTPS3* | AtTPS3-SP1 | AGTGAACTCAATGACGCTTTCGATG |
|  |  | AtTPS3-SP2 | TCCCAAAATATGACCAATTTCTGGTT |
|  | *AtTPS4* | AtTPS4-SP1 | GTAACGATGCAACCATAACATACCAGTC |
|  |  | AtTPS4-SP2 | CCCACCAAAATCGTTTTTGAACTTC |
|  | *AtTPS5* | AtTPS5-SP1 | GGTGCAGCCAGGTTCCATTAGG |
|  |  | AtTPS5-SP2 | CGTTGTTAGAAGCCTCTCTGCCAC |
|  | *AtTPS6* | AtTPS6-SP1 | CTAAAAGCCGAGAAACTCTATCGGACT |
|  |  | AtTPS6-SP2 | TCATCGAAAGCATCCTTCTCGCTA |

**Table S3.** Continued.

| **Plants** | **Gene** | **Primer name** | **Sequence（5’-3’）** |
| --- | --- | --- | --- |
| *Arabidopsis* | *AtTPS7* | AtTPS7-SP1 | TGGAAGAGGGAGGGAAAGTTTAAGC |
|  |  | AtTPS7-SP2 | AACCGGTTTTCCCTTTCCGG |
|  | *AtTPS8* | AtTPS8-SP1 | TCCAAGCAACGAGGTTGTCTCTGT |
|  |  | AtTPS8-SP2 | TCTCGGATTACTTTCTCCGCAGC |
|  | *AtTPS9* | AtTPS9-SP1 | ATTCGTCGGCTGAGGCGGA |
|  |  | AtTPS9-SP2 | AAAGATCTCAGGTGGCATTGGGA |
|  | *AtTPS10* | AtTPS10-SP1 | TGTTAGCGGGAGGGGAAAAGTT |
|  |  | AtTPS10-SP2 | CGGCTAAGAATTTTCCCGGTGA |
|  | *AtTPS11* | AtTPS11-SP1 | AGCAAATGGTTTGACTCTTGCCC |
|  |  | AtTPS11-SP2 | TGCTATCAAATGCTCCACCACCTT |
|  | *Actin* | AtActin-CL1 | ATGAAGATTAAGGTCGTGGCAC |
|  |  | AtActin-CL2 | GTTTTTATCCGAGTTTGAAGAGGC |
| Rice | *OsTPS1* | OsTPS1-SP3 | CTCGTCGTCGCCAACCGC |
|  |  | OsTPS1-SP4 | TTGCTCGCTTGTACGCGTTGA |
|  | *OsTPS2* | OsTPS2-SP1 | AGACGCAGGGACAAATTGGGAG |
|  |  | OsTPS2-SP2 | AAGTCAGCCTGCTTTCCTCTCTCC |
|  | *OsTPS3* | OsTPS3-SP1 | CGAGGAAGGGATAGCCTTGAGAAA |
|  |  | OsTPS3-SP2 | AATCTGCCTGTCTCTTGTTCTCTGTCA |
|  | *OsTPS4* | OsTPS4-SP1 | TTCGAGAAGGGAACACTCCATGAC |
|  |  | OsTPS4-SP2 | AGGTGGAGCTTGTGATCATCTGGA |
|  | *OsTPS5* | OsTPS5-SP1 | GAGGGAAGGATGAACTTGGCAGA |
|  |  | OsTPS5-SP2 | CGCCTTCCCTCGGCTCAC |
|  | *OsTPS6* | OsTPS6-SP1 | AAGGATGACTTGAGCAAAAAGCTTATCT |
|  |  | OsTPS6-SP2 | TACAAAATCTGCCAGTTGCCCCT |
|  | *OsTPS7* | OsTPS7-SP1 | AAGAGCGCGTAGTTTGCTTGATGA |
|  |  | OsTPS7-SP2 | CCGTTGTTCATCAATCTGTGAATTACC |
|  | *OsTPS8* | OsTPS8-SP1 | TCATGCAAGTTGGTGACACATTTTAAT |
|  |  | OsTPS8-SP2 | AAGATCTGTGCAGTCTCCGGGAA |
|  | *OsTPS9* | OsTPS9-SP1 | GCAAGGACGACCTCGAACGC |
|  |  | OsTPS9-SP2 | CATGGCCGACACGATGCACTC |
|  | *OsTPS10* | OsTPS10-SP1 | TGAGCACAAAGAAGCGGTCGACT |
|  |  | OsTPS10-SP2 | GACAAAGTCCGGAAGGAGGCTG |
|  | *OsTPS11* | OsTPS11-SP1 | CAGCGGAAGAGATCGTACTACCCTAAG |
|  |  | OsTPS11-SP2 | GTTTTCGTTGTTGATTACCGTCCG |
|  | *Actin* | OsActin-3 | GTCTGCGATAATGGAACTGGTA |
|  |  | OsActin-4 | CAGGGCGATGTAGGAAAGC |
